# (*E*)-2-(2-Hy­droxy-3-methyl­benzyl­idene)-*N*-methyl­hydrazine-1-carbo­thio­amide: supra­molecular assemblies in two-dimensions mediated by N—H⋯S and C—H⋯π inter­actions

**DOI:** 10.1107/S2056989019004444

**Published:** 2019-04-05

**Authors:** Md. Azharul Arafath, Huey Chong Kwong, Farook Adam

**Affiliations:** a Department of Chemistry, Shahjalal University of Science and Technology, Sylhet 3114, Bangladesh; b School of Chemical Sciences, Universiti Sains Malaysia, Penang 11800 USM, Malaysia

**Keywords:** crystal structure, hydrazine carbo­thio­amide, Schiff base, inter­molecular inter­action

## Abstract

In the title compound, C_10_H_13_N_3_OS, the azomethine C=N double bond has an *E* configuration. The phenyl ring and methyl­hydrazine carbo­thio­amide moiety [maximum deviation = 0.008 (2) Å] are twisted slightly with a dihedral angle of 14.88 (10)°. In the crystal, mol­ecules are linked into sheets parallel to the *ab* plane *via* N—H⋯S hydrogen bonds and C—H⋯π inter­actions.

## Abstract 2

The character of the methyl­hydrazine carbo­thio­amide moiety in the title compound is a thio­semicarbazone Schiff base was confirmed by its bond lengths and bond angles. In the crystal, mol­ecules of the title compound are mediated into sheets parallel to the *ab* plane by N—H⋯S hydrogen bonds and C—H⋯π inter­actions.

## Chemical context   

Schiff base compounds are very important and can be used for multidisciplinary applications. They are widely used in the food and dye industries and exhibit many types of biological activity (Gaur, 2000 ▸) such as anti­bacterial, anti­fungal, and anti­malarial (Annapoorani & Krishnan, 2013 ▸). The azomethine C=N group of Schiff bases plays an important role in the biological activity. Metal complexes of thio­semicarbazones have also received much attention. The metal chelation typically improves the lipophilicity of the ligand and facilitates the penetration of the complexes into bacterial membranes (Lobana *et al.*, 2009 ▸; Rogolino *et al.*, 2017 ▸). Thio­semi­carbazones have multi-donor characteristics because of the presence of nitro­gen and sulfur atoms in their mol­ecular backbone. This results in a variety of coordination modes and many different physiochemical properties (Sharma *et al.*, 2016 ▸). As part of our ongoing studies on thio­semicarbazone Schiff bases (Arafath *et al.*, 2018*a* ▸), we report herein the synthesis and structural determination of the title compound.
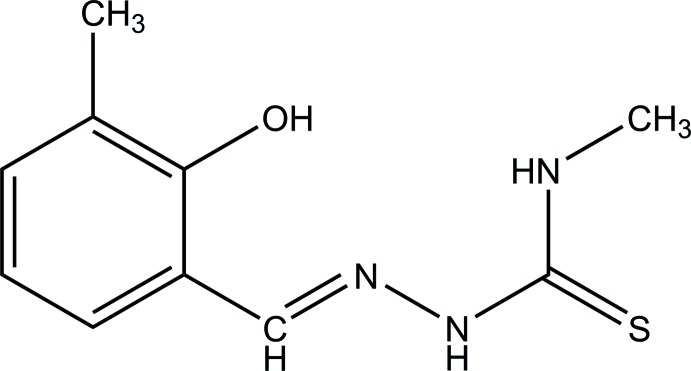


## Structural commentary   

**Figure 1:**
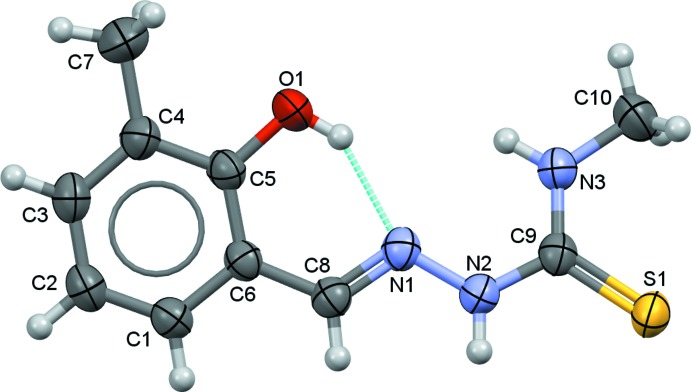
The atom labelling scheme and displacement ellipsoids of the mol­ecular structure at the 50% probability level.

**Figure 2:**
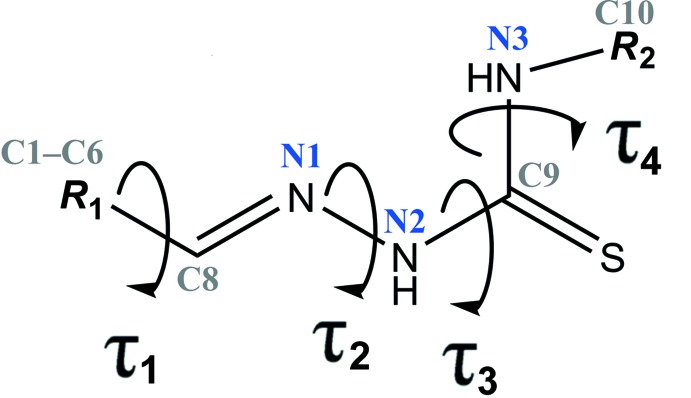
General chemical diagram showing torsion angles, τ_1_, τ_2_, τ_3_ and τ_4_ in the title compound.

The title compound (I) crystallizes in the non-centrosymmetric ortho­rhom­bic space group *Iba*2 and exhibits an *E* configuration with respect to the azomethine C=N double bond (Fig. 1 ▸). The C8=N1 and C9=S1 bond lengths of 1.288 (3) and 1.689 (2) Å, respectively, confirm the presence of the double bonds while the C6—C8, N2—C9 and C9—N3 bond lengths of 1.452 (3), 1.354 (3) and 1.321 (3) Å, respectively, confirm their single-bond character. The C6—C8—N1 and N2—C9—N3 angles are 122.5 (2) and 117.8 (2)°, respectively, and are consistent with an *sp* ^2^-hybridized character for atom C8 and C9 (Arafath *et al.*, 2018*b* ▸; Khalaji *et al.*, 2012 ▸). The unique mol­ecular conformation of (I) can be characterized by four torsion angles, *viz. τ*_1_ (C5—C6—C8—N1), *τ*_2_ (C8—N1—N2—C9), *τ*_3_ (N1—N2—C9—N3) and *τ*_4_ (N2—C9—N3—C10), respectively (Fig. 2 ▸). The torsion angles *τ*_3_ and *τ*_4_ are 0.4 (3) and 179.9 (2)°, signifying the planarity of the methyl­hydrazine carbo­thio­amide moiety [N1—N2—(C9=S1)—N3—C10; mean deviation σ = 0.002 Å, maximum deviation = 0.008 (2) Å for atom C9]. *τ*_1_ and *τ*_2_ are slightly twisted [*τ*_1_ = −4.2 (3) and *τ*_2_ = 170.4 (2)°, respectively], and the C1–C6 phenyl ring and the methyl­hydrazine carbo­thio­amide moiety subtend a dihedral angle of 14.88 (10)°. In the mol­ecule, the hy­droxy group acts as a hydrogen-bond donor for the adjacent hydrazine group, forming a intra­molecular hydrogen bond with an *S*(6) ring motif (Fig. 1 ▸, Table 1 ▸).

## Supra­molecular features   

**Figure 3:**
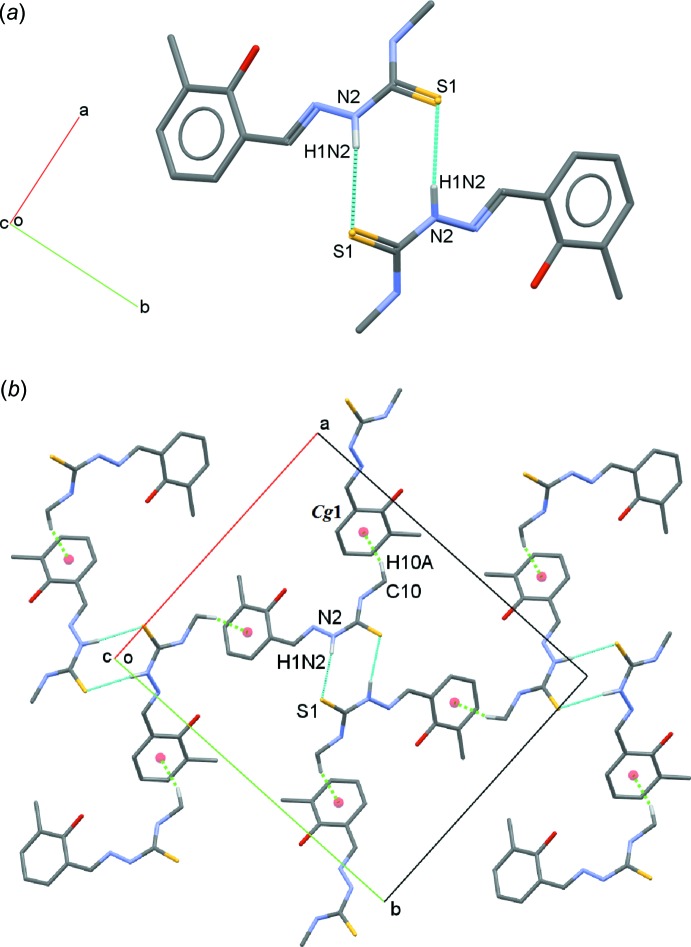
(*a*) A view of a dimer of C_10_H_13_N_3_OS with N2—H1*N*2⋯S1 hydrogen bonds shown as cyan dotted lines. (*b*) A view of a dimeric sheet with C10—H10*A*⋯*Cg*1 inter­actions shown as green dotted lines. Hydrogen atoms not involved in with these inter­actions are omitted for clarity.

In the crystal, mol­ecules are linked into dimers with an 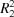
(8) ring motif *via* N2—H1*N*2⋯S1 hydrogen bonds (Fig. 3 ▸ *a*, Table 1 ▸). The dimers are connected into sheets parallel to the *ab* plane through C—H⋯π inter­actions (Fig. 3 ▸ *b*, Table 1 ▸).

## Database survey   

A search of the Cambridge Structural Database (CSD version 5.39, last update February 2018; Groom *et al.*, 2016 ▸) using (*E*)-2-(2-hy­droxy­benzyl­idene)-*N*-(λ^1^-meth­yl)hydrazine-1-carbo­thio­amide as reference moiety found 44 structures containing the 2-(2-hy­droxy­benzyl­idene)hydrazinecarbo­thio­amide moiety with different substituents. The basic structural motif (*E*)-2-(2-hy­droxy­benzyl­idene)-*N*-(λ^1^-meth­yl)hydrazine-1-carbo­thio­amide is shown in Fig. 2 ▸ and the different substit­uents (***R*** **_1_** and ***R*** **_2_**) together with the torsion angles of the C—CH=N—NH—C(=S)—NH—C backbone are summarized in Table 2 ▸. In these structures, the torsion angle τ_1_ exists in either the *syn-periplanar* (range from 0 to 12°) or *anti-periplanar* (range from 167 to 179°) conformation. As for the torsion angle τ_2_, all structures adopt an *anti-periplanar* conformation (169–179°). Similar to the title compound, torsion angles τ_3_ and τ_4_ for most of the structures are *syn-periplanar* (0–16°) and *anti-periplanar* (171-180°), respectively. However, there are two outliers (YOCJOR and YOCJUX; (Chumakov *et al.*, 2014 ▸)) where the 2-(2-hy­droxy­benzyl­idene) hydrazinecarbo­thio­amide is substituted with a pyridine ring. In contrast to most of the structures, torsion angles τ_3_ and τ_4_ for YOCJOR and YOCJUX are *anti-periplanar* (178 and 177°, respectively) and *syn-periplanar* (1 and 3°, respectively).

## Synthesis and crystallization   

**Figure 4:**
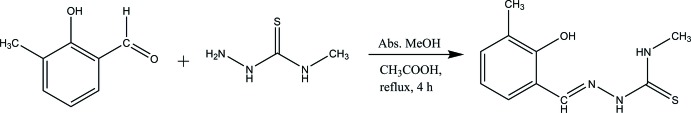
Reaction scheme for the synthesis of C_10_H_13_N_3_OS.

2-Hy­droxy-3-methyl­benzaldehyde (0.68 g, 5.00 mmol) was dissolved in 20.0 mL of methanol. 0.20 mL of glacial acetic acid was added and the mixture was refluxed for 30 minutes. A solution of 0.52 g (5.00 mmol) of *N*-methyl hydrazinecarbo­thio­amide in 20.0 mL of methanol was added dropwise with stirring to the aldehyde solution (Fig. 4 ▸). The resulting colourless solution was heated under reflux for 4 h with stirring. The crude product was washed with 5.0 mL of *n*-hexane. The recovered product was dissolved in DMSO for purification and recrystallization. Light-yellow single crystals (m.p. 454–455 K; yield 94%) suitable for X-ray diffraction were obtained by slow evaporation of the solvent.

Analysis calculated for C_10_H_13_N_3_OS (FW: 223.29 g mol^−1^); C, 53.74; H, 5.83; N, 18.81; found: C, 53.71; H, 5.79; N, 18.83%. ^1^H NMR (500 MHz, DMSO-*d*_6_, Me_4_Si ppm): δ 11.38 (*s*, N—NH), δ 9.39 (*s*, OH), δ 8.34 (*s*, HC=N), δ 8.44 (*q*, CS–NH), δ 7.42–6.81 (multiplet, aromatic), δ 3.00 (*d*, *J* = 4.5 Hz, N—CH_3_), δ 2.20 (*s*, Ph—CH_3_). ^13^C NMR (DMSO-*d*_6_, Me_4_Si ppm): δ 177.48 (C=S), δ 154.24 (C=N), δ 143.64–119.10 (C-aromatic), δ 31.05 (N—CH_3_), δ 15.91(Ph—CH_3_) ppm. IR (KBr pellets υ_max_/cm^−1^): 3418 υ(NH), 3133 υ(OH), 2983(NC—H_3_, *sp^3^*), 1618 υ(C=N), 1553 υ(C=C, aromatic), 1270 υ(C=S), 1251 υ(CH, bend., aromatic), 1085 υ(C—O). 1043 υ(C—N).

## Refinement   

Crystal data, data collection and structure refinement details are summarized in Table 3 ▸. C-bound H atoms were positioned geometrically (C—H = 0.93–0.96 Å) and refined using a riding model with *U*_iso_(H) = 1.2 or 1.5 *U*_eq_(C). All N- and O-bound H atoms were located from a difference-Fourier map and freely refined.

## Supplementary Material

Crystal structure: contains datablock(s) I. DOI: 10.1107/S2056989019004444/nr2074sup1.cif

Structure factors: contains datablock(s) I. DOI: 10.1107/S2056989019004444/nr2074Isup2.hkl

Supporting information file. DOI: 10.1107/S2056989019004444/nr2074Isup3.cml

CCDC reference: 1485713

Additional supporting information:  crystallographic information; 3D view; checkCIF report

## Figures and Tables

**Table 1:** Hydrogen-bond geometry (Å, °) *Cg*1 is the centroid of the C1–C6 phenyl ring.

*D*—H⋯*A*	*D*—H	H⋯*A*	*D*⋯*A*	*D*—H⋯*A*
O1—H1*O*1⋯N1	0.84 (4)	1.94 (4)	2.681 (3)	147 (4)
N2—H1*N*2⋯S1^i^	0.89 (3)	2.51 (3)	3.387 (2)	173 (3)
C10—H10*A*⋯*Cg*1^ii^	0.96	2.70	3.577 (4)	152

Symmetry codes: (i) 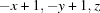; (ii) 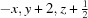.

**Table 2:** Torsion angles τ_1_, τ_2_, τ_3_ and τ_4_ (°)

Compound	*R*_1_	*R*_2_	τ_1_	τ_2_	τ_3_	τ_4_
(I)	2-hy­droxy-3-methyl­benzyl­iden­yl	meth­yl	4	170	0	180
AWAZOP (Hussein & Guan, 2015 ▸)	5-bromo-2-hy­droxy­benzyl­iden­yl	meth­yl	1	175	12	179
AWEBEL (Hussein & Guan, 2015 ▸)	3-eth­oxy-2-hy­droxy­benzyl­iden­yl	meth­yl	176	174	4	180
CIVZAK (Hussein *et al.*, 2014*b* ▸)	5-(*tert*-but­yl)-2-hy­droxy­benzyl­iden­yl	eth­yl	2	174	15	180
CIWBAN (Hussein *et al.*, 2014*b* ▸)	5-allyl-3-ethyl-2-hy­droxy­benzyl­iden­yl	meth­yl	169	173	5	178
DAGVOZ (Arafath *et al.*, 2017*b* ▸)	2-hy­droxy-5-meth­oxy-3-nitro­benzyl­iden­yl	meth­yl	177	176	7	179
EFUPAX (Rubčić *et al.*, 2008 ▸)	2-hy­droxy-4-meth­oxy­benzyl­iden­yl	phen­yl	2	173	4	174
EROVIR (Lo & Ng, 2011 ▸)	5-chloro-2-hy­droxy­benzyl­iden­yl	eth­yl	8	172	14	176
GOZQIX (Hussein *et al.*, 2015*a* ▸)	2-hy­droxy-5-meth­oxy­benzyl­iden­yl	meth­yl	3	175	14	180
GOZQIX01 (Salam *et al.*, 2016 ▸)	2-hy­droxy-5-meth­oxy­benzyl­iden­yl	meth­yl	3	175	15	180
GOZQIX02 (Subhashree *et al.*, 2017 ▸)	2-hy­droxy-5-meth­oxy­benzyl­iden­yl	meth­yl	2	175	13	180
HABDEW (Hussein *et al.*, 2015*c* ▸)	3-eth­oxy-2-hy­droxy­benzyl­iden­yl	eth­yl	177	176	5	180
HABFEY (Hussein *et al.*, 2015*c* ▸)	5-allyl-2-hy­droxy-3-meth­oxy­benzyl­iden­yl	eth­yl	173, 173	176, 179	6, 8	178, 177
HAXROO (Vrdoljak *et al.*, 2005 ▸)	2-hy­droxy­benzyl­iden­yl	meth­yl	1	176	11	178
HAXROO01 (Liu, 2015 ▸)	2-hy­droxy­benzyl­iden­yl	meth­yl	2	175	11	178
HAXSAB (Vrdoljak *et al.*, 2005 ▸)	2-hy­droxy-3-meth­oxy­benzyl­iden­yl	meth­yl	177	174	5	178
IBAZUJ (Haque *et al.*, 2015 ▸)	2,3-di­hydroxy­benzyl­iden	meth­yl	1	170	1	175
IBEDOL (Haque *et al.*, 2015 ▸)	2-hy­droxy-5-methyl­benzyl­iden­yl	meth­yl	3, 2	175, 173	16, 16	175, 175
IFUXEN (Tan *et al.*, 2008*b* ▸)	2,4-di­hydroxy­benzyl­iden­yl	eth­yl	2	179	0	176
IFUXEN01 (Hussein *et al.*, 2014*b* ▸)	2,4-di­hydroxy­benzyl­iden­yl	eth­yl	2	179	0	176
IFUXEN02 (Ramaiyer & Frank, 2015 ▸)	2,4-di­hydroxy­benzyl­iden­yl	eth­yl	1	175	4	179
IFUXEN03 (Ramaiyer & Frank, 2015 ▸)	2,4-di­hydroxy­benzyl­iden­yl	eth­yl	5	171	6	178
IGALUY (Tan *et al.*, 2008*c* ▸)	2,4-di­hydroxy­benzyl­iden­yl	meth­yl	5	174	9	176
IGALUY01 (Salam *et al.*, 2015 ▸)	2,4-di­hydroxy­benzyl­iden­yl	meth­yl	2	177	16	178
IMAFIN (El-Asmy *et al.*, 2016 ▸)	2-hy­droxy­benzyl­iden­yl	eth­yl	1	177	13	177
JAJHUA (Li *et al.*, 2016 ▸)	5-bromo-2-hy­droxy­benzyl­iden­yl	meth­yl	1	175	12	179
JOFHIW (Tan *et al.*, 2008*a* ▸)	2,5-di­hydroxy­benzyl­iden	meth­yl	1	175	11	178
KOCLIY (Đilović *et al.*, 2008 ▸)	4-(di­ethyl­amino)-2-hy­droxy­benzyl­iden­yl	phen­yl	2	172	12	174
LAQCIR (Jacob & Kurup, 2012 ▸)	5-bromo-2-hy­droxy-3-meth­oxy­benzyl­iden­yl	cyclo­hex­yl	172	177	4	179
NUQNAP (Shawish *et al.*, 2010 ▸)	2,3,4-tri­hydroxy­benzyl­iden­yl	eth­yl	167	176	8	174
OBOLOJ (Arafath *et al.*, 2017*a* ▸)	5-chloro-2-hy­droxy­benzyl­iden­yl	cyclo­hex­yl	175	176	6	177
PAXCAU (Jacob *et al.*, 2012 ▸)	5-bromo-2-hy­droxy-3-meth­oxy­benzyl­iden­yl	phen­yl	177	180	6	177
RIVFAE (Seena *et al.*, 2008 ▸)	2-hy­droxy­benzyl­iden­yl	phen­yl	2, 5, 2	179, 175, 178	12, 9, 2	171, 177, 180
RIVFAE01 (Rubcic *et al.*, 2008 ▸)	2-hy­droxy­benzyl­iden­yl	phen­yl	11, 3	177, 171	2, 2	175, 170
SUKQOG (Hussein *et al.*, 2015*d* ▸)	5-allyl-2-hy­droxy-3-meth­oxy­benzyl­iden­yl	phen­yl	168	172	4	179
WEXDAG (Orysyk *et al.*, 2013 ▸)	2-hy­droxy­benzyl­iden­yl	all­yl	4	170	7	173
XOTPED (Hussein *et al.*, 2015*b* ▸)	2-hy­droxy-3-methyl­benzyl­iden­yl	eth­yl	2	179	7	179
YOCJOR (Chumakov *et al.*, 2014 ▸)	5-bromo-2-hy­droxy­benzyl­iden­yl	pyridin-2-yl	0	179	178	1
YOCJUX (Chumakov *et al.*, 2014 ▸)	2-hy­droxy-3-meth­oxy­benzyl­iden­yl	pyridin-2-yl	3	178	177	3
YOPHUI (Hussein *et al.*, 2014*a* ▸)	3-(*tert*-but­yl)-2-hy­droxy­benzyl­iden­yl	eth­yl	4, 8	171, 169	4, 18	179, 180
YOPLIA (Hussein *et al.*, 2014*a* ▸)	2-hy­droxy-5-methyl­benzyl­iden­yl	eth­yl	4	171	10	180
YUKYOU (Salam & Haque, 2015 ▸)	3,5-di­chloro-2-hy­droxy­benzyl­iden­yl	eth­yl	179	180	2	178
YUXJOS (Arafath *et al.*, 2018*a* ▸)	3-(*tert*-but­yl)-2-hy­droxy­benzyl­iden­yl	cyclo­hex­yl	12	170	12	176
ZIJKIO (Li & Sato, 2013 ▸)	5-bromo-2-hy­droxy­benzyl­iden­yl	eth­yl	6	172	12	176
ZIJKIO02 (Hussein *et al.*, 2015*b* ▸)	5-bromo-2-hy­droxy­benzyl­iden­yl	eth­yl	7	173	13	177

Note: there is more than one torsion angle for compounds HABFEY, IBEDOL, RIVFAE, RIVFAE01 and YOPHUI because there are more than one independent mol­ecules in their asymmetric units.

**Table 3:** Experimental details

Crystal data	
Chemical formula	C_10_H_13_N_3_OS
*M*_r_	223.29
Crystal system, space group	Orthorhombic, *I* *b* *a*2
Temperature (K)	296
*a*, *b*, *c* (Å)	14.6474 (14), 17.522 (2), 8.9048 (8)
*V* (Å^3^)	2285.4 (4)
*Z*	8
Radiation type	Mo *K*α
μ (mm^−1^)	0.26
Crystal size (mm)	0.46 × 0.26 × 0.16

Data collection	
Diffractometer	Bruker APEXII DUO CCD area-detector
Absorption correction	Multi-scan (*SADABS*; Bruker, 2012 ▸)
*T*_min_, *T*_max_	0.853, 0.879
No. of measured, independent and observed [*I* > 2σ(*I*)] reflections	14825, 3359, 2949
*R*_int_	0.020
(sin θ/λ)_max_ (Å^−1^)	0.705

Refinement	
*R*[*F* ^2^ > 2σ(*F* ^2^)], *wR*(*F* ^2^), *S*	0.033, 0.094, 1.06
No. of reflections	3359
No. of parameters	150
No. of restraints	1
H-atom treatment	H atoms treated by a mixture of independent and constrained refinement
Δρ_max_, Δρ_min_ (e Å^−3^)	0.17, −0.16
Absolute structure	Flack parameter determined using 1222 quotients [(*I* ^+^)−(*I* ^−^)]/[(*I* ^+^)+(*I* ^−^)] (Parsons *et al.*, 2013 ▸)
Absolute structure parameter	0.04 (3)

Computer programs: *APEX2* and *SAINT* (Bruker, 2012 ▸), *SHELXS97* (Sheldrick, 2008 ▸), *SHELXL2013* (Sheldrick, 2015 ▸), *Mercury* (Macrae *et al.*, 2006 ▸) and *PLATON* (Spek, 2009 ▸).

## supplementary crystallographic information

### Crystal data

**Table:**

C_10_H_13_N_3_OS	*D*_x_ = 1.298 Mg m^−^^3^
*M**_r_* = 223.29	Mo *K*α radiation, λ = 0.71073 Å
Orthorhombic, *I**b**a*2	Cell parameters from 5563 reflections
*a* = 14.6474 (14) Å	θ = 2.3–29.5°
*b* = 17.522 (2) Å	µ = 0.26 mm^−^^1^
*c* = 8.9048 (8) Å	*T* = 296 K
*V* = 2285.4 (4) Å^3^	Block, yellow
*Z* = 8	0.46 × 0.26 × 0.16 mm
*F*(000) = 944	

### Data collection

**Table:**

Bruker APEXII DUO CCD area-detector diffractometer	3359 independent reflections
Radiation source: fine-focus sealed tube	2949 reflections with *I* > 2σ(*I*)
Graphite monochromator	*R*_int_ = 0.020
φ and ω scans	θ_max_ = 30.1°, θ_min_ = 1.8°
Absorption correction: multi-scan (SADABS; Bruker, 2012)	*h* = −20→20
*T*_min_ = 0.853, *T*_max_ = 0.879	*k* = −23→24
14825 measured reflections	*l* = −12→12

### Refinement

**Table:**

Refinement on *F*^2^	Hydrogen site location: mixed
Least-squares matrix: full	H atoms treated by a mixture of independent and constrained refinement
*R*[*F*^2^ > 2σ(*F*^2^)] = 0.033	*w* = 1/[σ^2^(*F*_o_^2^) + (0.0497*P*)^2^ + 0.3888*P*] where *P* = (*F*_o_^2^ + 2*F*_c_^2^)/3
*wR*(*F*^2^) = 0.094	(Δ/σ)_max_ = 0.001
*S* = 1.06	Δρ_max_ = 0.17 e Å^−^^3^
3359 reflections	Δρ_min_ = −0.15 e Å^−^^3^
150 parameters	Absolute structure: Flack parameter determined using 1222 quotients [(*I*^+^)-(*I*^-^)]/[(*I*^+^)+(*I*^-^)] (Parsons *et al.*, 2013)
1 restraint	Absolute structure parameter: 0.04 (3)

### Special details

**Table:**

Geometry. All esds (except the esd in the dihedral angle between two l.s. planes) are estimated using the full covariance matrix. The cell esds are taken into account individually in the estimation of esds in distances, angles and torsion angles; correlations between esds in cell parameters are only used when they are defined by crystal symmetry. An approximate (isotropic) treatment of cell esds is used for estimating esds involving l.s. planes.

### Fractional atomic coordinates and isotropic or equivalent isotropic displacement parameters (Å^2^)

**Table:**

	*x*	*y*	*z*	*U*_iso_*/*U*_eq_	
S1	0.35594 (4)	0.50006 (3)	0.20442 (10)	0.05018 (16)	
O1	0.45729 (12)	0.77741 (11)	0.6162 (3)	0.0621 (6)	
N1	0.48391 (12)	0.65142 (9)	0.4511 (3)	0.0414 (4)	
N2	0.46275 (13)	0.59332 (10)	0.3526 (2)	0.0436 (4)	
N3	0.31035 (13)	0.60707 (11)	0.4029 (2)	0.0459 (4)	
C1	0.69785 (15)	0.72085 (14)	0.6081 (2)	0.0443 (5)	
H1A	0.735599	0.683585	0.567182	0.053*	
C2	0.73506 (15)	0.77658 (13)	0.6987 (3)	0.0493 (5)	
H2A	0.797160	0.776261	0.720393	0.059*	
C3	0.67936 (16)	0.83287 (14)	0.7568 (3)	0.0492 (5)	
H3A	0.704945	0.871242	0.815398	0.059*	
C4	0.58596 (16)	0.83349 (12)	0.7297 (3)	0.0479 (5)	
C5	0.54896 (14)	0.77591 (12)	0.6405 (3)	0.0424 (4)	
C6	0.60427 (14)	0.71939 (12)	0.5768 (2)	0.0380 (4)	
C7	0.5255 (2)	0.89533 (18)	0.7931 (5)	0.0783 (10)	
H7A	0.491901	0.918970	0.713173	0.117*	
H7B	0.562565	0.932944	0.842504	0.117*	
H7C	0.483746	0.873420	0.864103	0.117*	
C8	0.56962 (15)	0.65960 (11)	0.4793 (3)	0.0410 (4)	
H8A	0.610876	0.625925	0.435625	0.049*	
C9	0.37548 (14)	0.57095 (11)	0.3289 (2)	0.0397 (4)	
C10	0.21430 (16)	0.58990 (19)	0.3892 (4)	0.0647 (7)	
H10A	0.179253	0.629791	0.435460	0.097*	
H10B	0.201496	0.542285	0.438227	0.097*	
H10C	0.198298	0.586205	0.284940	0.097*	
H1N2	0.5065 (18)	0.5680 (16)	0.307 (4)	0.056 (8)*	
H1N3	0.3243 (19)	0.6428 (16)	0.471 (4)	0.054 (7)*	
H1O1	0.444 (3)	0.7429 (19)	0.555 (5)	0.076 (10)*	

### Atomic displacement parameters (Å^2^)

**Table:**

	*U*^11^	*U*^22^	*U*^33^	*U*^12^	*U*^13^	*U*^23^
S1	0.0523 (3)	0.0455 (3)	0.0527 (3)	−0.0030 (2)	−0.0095 (3)	−0.0097 (2)
O1	0.0328 (8)	0.0601 (11)	0.0936 (16)	−0.0015 (7)	−0.0014 (8)	−0.0230 (10)
N1	0.0439 (9)	0.0367 (7)	0.0436 (8)	−0.0042 (6)	−0.0024 (9)	0.0006 (9)
N2	0.0412 (9)	0.0404 (9)	0.0491 (10)	0.0001 (7)	−0.0030 (8)	−0.0060 (8)
N3	0.0409 (9)	0.0495 (10)	0.0475 (9)	0.0000 (8)	−0.0055 (7)	−0.0078 (8)
C1	0.0380 (10)	0.0490 (12)	0.0460 (11)	0.0012 (9)	0.0008 (9)	0.0009 (9)
C2	0.0379 (9)	0.0576 (12)	0.0525 (11)	−0.0059 (9)	−0.0058 (10)	0.0016 (12)
C3	0.0478 (12)	0.0485 (11)	0.0513 (11)	−0.0119 (10)	−0.0057 (10)	−0.0017 (9)
C4	0.0446 (11)	0.0416 (10)	0.0575 (15)	−0.0049 (9)	0.0020 (9)	−0.0064 (9)
C5	0.0327 (10)	0.0401 (10)	0.0544 (12)	−0.0048 (8)	0.0031 (8)	0.0002 (9)
C6	0.0357 (9)	0.0380 (10)	0.0401 (10)	−0.0037 (8)	0.0010 (8)	0.0033 (8)
C7	0.0642 (17)	0.0641 (17)	0.107 (3)	0.0081 (14)	−0.0021 (18)	−0.0325 (17)
C8	0.0413 (10)	0.0387 (9)	0.0428 (11)	−0.0008 (8)	−0.0006 (8)	0.0020 (8)
C9	0.0439 (10)	0.0365 (9)	0.0387 (9)	−0.0016 (8)	−0.0064 (8)	0.0033 (8)
C10	0.0403 (12)	0.0802 (18)	0.0736 (17)	−0.0043 (12)	−0.0015 (12)	−0.0148 (15)

### Geometric parameters (Å, º)

**Table:**

S1—C9	1.689 (2)	C2—H2A	0.9300
O1—C5	1.360 (3)	C3—C4	1.389 (3)
O1—H1O1	0.84 (4)	C3—H3A	0.9300
N1—C8	1.288 (3)	C4—C5	1.393 (3)
N1—N2	1.379 (3)	C4—C7	1.509 (4)
N2—C9	1.354 (3)	C5—C6	1.400 (3)
N2—H1N2	0.88 (3)	C6—C8	1.452 (3)
N3—C9	1.321 (3)	C7—H7A	0.9600
N3—C10	1.444 (3)	C7—H7B	0.9600
N3—H1N3	0.90 (3)	C7—H7C	0.9600
C1—C2	1.379 (3)	C8—H8A	0.9300
C1—C6	1.399 (3)	C10—H10A	0.9600
C1—H1A	0.9300	C10—H10B	0.9600
C2—C3	1.381 (4)	C10—H10C	0.9600
			
C5—O1—H1O1	108 (3)	C4—C5—C6	121.21 (19)
C8—N1—N2	115.18 (18)	C1—C6—C5	118.25 (19)
C9—N2—N1	121.68 (18)	C1—C6—C8	118.35 (19)
C9—N2—H1N2	118.0 (19)	C5—C6—C8	123.40 (18)
N1—N2—H1N2	120.2 (19)	C4—C7—H7A	109.5
C9—N3—C10	124.2 (2)	C4—C7—H7B	109.5
C9—N3—H1N3	120.5 (18)	H7A—C7—H7B	109.5
C10—N3—H1N3	115.1 (18)	C4—C7—H7C	109.5
C2—C1—C6	121.1 (2)	H7A—C7—H7C	109.5
C2—C1—H1A	119.4	H7B—C7—H7C	109.5
C6—C1—H1A	119.4	N1—C8—C6	122.5 (2)
C1—C2—C3	119.4 (2)	N1—C8—H8A	118.7
C1—C2—H2A	120.3	C6—C8—H8A	118.7
C3—C2—H2A	120.3	N3—C9—N2	117.8 (2)
C2—C3—C4	121.5 (2)	N3—C9—S1	123.86 (17)
C2—C3—H3A	119.3	N2—C9—S1	118.37 (16)
C4—C3—H3A	119.3	N3—C10—H10A	109.5
C3—C4—C5	118.4 (2)	N3—C10—H10B	109.5
C3—C4—C7	121.2 (2)	H10A—C10—H10B	109.5
C5—C4—C7	120.3 (2)	N3—C10—H10C	109.5
O1—C5—C4	117.4 (2)	H10A—C10—H10C	109.5
O1—C5—C6	121.4 (2)	H10B—C10—H10C	109.5
			
C8—N1—N2—C9	170.4 (2)	O1—C5—C6—C1	−179.2 (2)
C6—C1—C2—C3	−1.4 (4)	C4—C5—C6—C1	1.7 (3)
C1—C2—C3—C4	1.9 (4)	O1—C5—C6—C8	1.1 (3)
C2—C3—C4—C5	−0.6 (3)	C4—C5—C6—C8	−178.0 (2)
C2—C3—C4—C7	−179.8 (3)	N2—N1—C8—C6	178.06 (19)
C3—C4—C5—O1	179.6 (2)	C1—C6—C8—N1	176.1 (2)
C7—C4—C5—O1	−1.1 (4)	C5—C6—C8—N1	−4.2 (3)
C3—C4—C5—C6	−1.2 (3)	C10—N3—C9—N2	179.9 (2)
C7—C4—C5—C6	178.0 (3)	C10—N3—C9—S1	1.2 (3)
C2—C1—C6—C5	−0.3 (3)	N1—N2—C9—N3	0.4 (3)
C2—C1—C6—C8	179.4 (2)	N1—N2—C9—S1	179.17 (16)

### Hydrogen-bond geometry (Å, º)

*Cg*1 is the centroid of the C1–C6 phenyl ring.

**Table:**

*D*—H···*A*	*D*—H	H···*A*	*D*···*A*	*D*—H···*A*
O1—H1*O*1···N1	0.84 (4)	1.94 (4)	2.681 (3)	147 (4)
N2—H1*N*2···S1^i^	0.89 (3)	2.51 (3)	3.387 (2)	173 (3)
C10—H10*A*···*Cg*1^ii^	0.96	2.70	3.577 (4)	152

Symmetry codes: (i) −*x*+1, −*y*+1, *z*; (ii) −*x*, *y*+2, *z*+1/2.
